# Fabrication and Actuation of Cu-Ionic Polymer Metal Composite

**DOI:** 10.3390/polym12020460

**Published:** 2020-02-17

**Authors:** Liang Yang, Dongsheng Zhang, Xining Zhang, Aifen Tian

**Affiliations:** 1 School of Mechanical Engineering, Xi’an Jiaotong University, Xi’an 710049, China; yangliang20070126@163.com (L.Y.); zhangxining@mail.xjtu.edu.cn (X.Z.); 2 School of Materials Science and Engineering, Xi’an University of Science and Technology, Xi’an 710054, China; azt5555@163.com

**Keywords:** ionic polymer metal composite, actuator, properties, length

## Abstract

In this study, Cu-Ionic polymer metal composites (Cu-IPMC) were fabricated using the electroless plating method. The properties of Cu-IPMC in terms of morphology, water loss rate, adhesive force, surface resistance, displacements, and tip forces were evaluated under direct current voltage. In order to understand the relationship between lengths and actuation properties, we developed two static models of displacements and tip forces. The deposited Cu layer is uniform and smooth and contains about 90% by weight of copper, according to the energy-dispersive X-ray spectroscopy (EDS) analysis data obtained. The electrodes adhere well (level of 5B) on the membrane, to ensure a better conductivity and improve the actuation performance. The penetration depth of needle-like electrodes can reach up to around 70 μm, and the structure shows concise without complex branches, to speed up the actuation. Overall the maximum displacement increased as the voltage increased. The applied voltage for the maximum force output is 8–9 V. The root mean square error (RMSE) and determination coefficient (DC) of the displacement and force models are 1.66 and 1.23, 0.96 and 0.86, respectively.

## 1. Introduction

Ionic polymer metal composites (IPMCs) are known as actuator with low mass, fexibility, biocompatibility and large deformation under low applied voltage [1,2,3,4]. IPMCs can produce large deflections at low voltage (<5 V), and generate a corresponding electrical response in the process of mechanical bending [5,6,7]. IPMCs have been used in different fields such as biomechanical and biomedical applications, robotics, flexible sensing and micro-electro-mechanical system (MEMS), as well as the aerospace and vehicles industry [8,9,10,11,12]. The IPMC is comprised of an ion-exchange-polymer membrane and two metal electrodes plated on the both sides of the Nafion membrane [13,14,15]. Its electromechanical mechanism is that hydrated metal cations inside the actuator move toward the cathode side when a small potential is applied to the electrodes, and the IPMC bends toward the anode side due to the volume difference [16,17,18].

Commonly, IPMC actuators are fabricated by electroless plating platinum (Pt) electrodes on Nafion membrane [19]. However, this approach is time consuming and expensive. In addition, the metal electrode cracks during operation, resulting in performance degradation. Other metals, such as gold (Au) [20], palladium (Pd) [21] and conductive materials such as grapheme [22], multi-walled carbon nanotubes (MWCNT) [23] and a series of polymers have also been used as electrodes. Active metal copper is rarely used in IPMC electrodes, mainly as an additional layer to an electrochemically stable platinum or gold electrode, which can improve the force and displacement of IPMC [24,25,26,27], and overcome the problem with the metal cracking [26]. However, the copper is used as electrode materials alone to reserch the performance of IPMC actuators was not discussed in detail. In this paper, Deposition of Cu coating on both sides of Nafion membrane to fabricate Cu-IPMC actuator by eletcroless plating method. The morphology, mechanical properties and actuation behavior of Cu-IPMCs were investigated. Scanning electron microscopy (SEM) and energy-dispersive X-ray spectroscopy (EDS) were used to investigate the dispersion and percentage of Cu particles on Nafion membrane. Water loss rate, adhesive force, and surface resistance were evaluated, and the effects of the lengths of the IPMCs on displacement and tip force were systematically investigated. Finally, the optimization software was used to establish a mathematical model of displacement and force, which laid a theoretical foundation for the later performance improvement of Cu-IPMC.

## 2. Experimental

### 2.1. Materials

Ethylenediamine tetraacetic acid disodium salt (C_10_H_14_N_2_O_8_Na_2_·2H_2_O, 99.0%), potassium tartrate (C_4_H_4_O_6_K2·1/2H_2_O, 99.0%), trisodium citrate dehydrate (C_6_H_5_Na_3_O_7_·2H_2_O, 99.1%), Copper sulfate pentahydrate (CuSO_4_·5H_2_O, 99.0%), formaldehyde (HCHO, 37 wt %), potassium ferricyanide trihydrate (K_4_[Fe(CN)_6_]·3H_2_O, 99.5%) and 2, 2-dipyridine (C_10_H_8_N_2_, 99.0%) were obtained from Tianjin Chemical Reagent Corp (Tianjin, China). Nafion 117 films with the thickness of 0.187 mm were obtained from DuPon (Shanghai, China). All chemicals were used as received without further purification.

### 2.2. Fabrication

We used Nafion-117 as the substrate membrane. The specific processes are as follows [24,26,28,29].

Pre-treatment: in order to increase the interfacial area and enhance adhesion between the Nafion membrane and electrodes, the surface of the Nafion was roughened using sandpaper, and ultrasonically cleaned in deionized (DI) water (40 min, 60 °C) then successively immersed in 2 mol/L hydrochloric acid (20 min, 60 °C) and DI water (30 min, 60 °C) to purify the ionomer membrane.

Ion adsorption: the pre-treated membranes were soaked in silver nitrate solution (AgNO_3_, 3 g/L, 100 mL) for 12 h.

Copper plating solution: Ethylenediamine tetraacetic acid disodium salt (C_10_H_14_N_2_O_8_Na_2_·2H_2_O, 48.8 g), potassium tartrate (C_4_H_4_O_6_K2·1/2H_2_O, 35.05 g) and trisodium citrate dehydrate (C_6_H_5_Na_3_O_7_·2H_2_O, 30 g) were dissolved in water (1500 mL) using a magnetic stirrer (Yuhua instrument Co., Ltd., Zhengzhou, China). Sodium hydroxide (NaOH) was added in order to raise the pH to 11. Copper sulfate pentahydrate (CuSO_4_·5H_2_O, 40.025 g), formaldehyde (HCHO, 37 wt %, 37.5 mL), potassium ferricyanide trihydrate (K_4_[Fe(CN)_6_]·3H_2_O, 0.025 g) and 2, 2-dipyridine (C_10_H_8_N_2_, 0.05 g) were added in the above solution, and kept stirring to make plating solution uniform.

Electroless plating copper: This process is essential to further increase the thickness of the surface electrode and decrease the surface resistivity. After being rinsed in deionized (DI) water, the membranes were immersed in plating solution (40 °C) for 35 min.

Ion exchange: the Cu-IPMC samples were immersed in an lithium chloride solution (LiCl) for 60 min to let the H+ ions of Nafion get replaced with small Li+ ions. Finally, they were preserved in deionized (DI) water.

After the preparation steps were finished, the IPMC was cut into the needed sizes and numbered, as shown in Table 1 (L represents length). The L4 with a length of 50 mm is used as an example for further testing and research.

### 2.3. Measurements

The cross-section and surface morphologies were observed by SEM (JEM 2010). The low-temperature cracking method was used to obtain the cross-sections so as to protect the electrodes from damage; during this procedure all samples were clamped by two tweezers, soaked in liquid nitrogen for 30 s and then broken into pieces. EDS data was measured by using Energy 300 Oxford instrument on the surface of the IPMCs to provide the Cu content. All the samples tested were freshly made.

The IPMC samples were soaked into DI water at room temperature for 24 h, to absorb a maximum amount of water. The water loss rate was gravimetrically determined with the ratio of the mass reduction in the wet to the mass in the dry. And these masses were measured by using electronic balance (FA2004) every 5 min.

The adhesive force of the Cu coating to the substrate was measured following the GB9286 standard. To scratch the grid (1 × 1 mm^2^) on the surface of IPMC by a knife, then tape (Scotch, 3M600) to paste tightly, and quickly peel off at an angle of 180 after about 5 min.

The newly prepared IPMCs were took out to prepare the test after fully absorbing water in deionized water to reach saturation state, and the excess water on the surface was wiped clean with filter paper. The IPMC is divided into five equal parts, with each part length of 10 mm. The surface resistance of IPMC sample was measured between two points at a distance of 10, 20, 30, 40, and 50 mm to determine the uniformity of the coating, and the average value over 3 measurements was adopted.

If the tip of sample was fixed on load cell, force was transferred to load cell by line contact and the middle of sample started to buckle and rised because there was no constraint to inhibit such a motion. So in this study, in which a surface contact was created between sample and balance, and length of surface contact was fixed as 30 mm for the sample length of 50 mm. The forces of samples were measured by precision balance (FA2004), with an accuracy of 0.0001g. Displacement of the IPMCs under different electrical stimuli was captured by a digital camera. All measurements were carried out in air at room temperature, and the average value over three measurements was adopted.

## 3. Results and Discussion

### 3.1. Morphology

**Figure 1:**
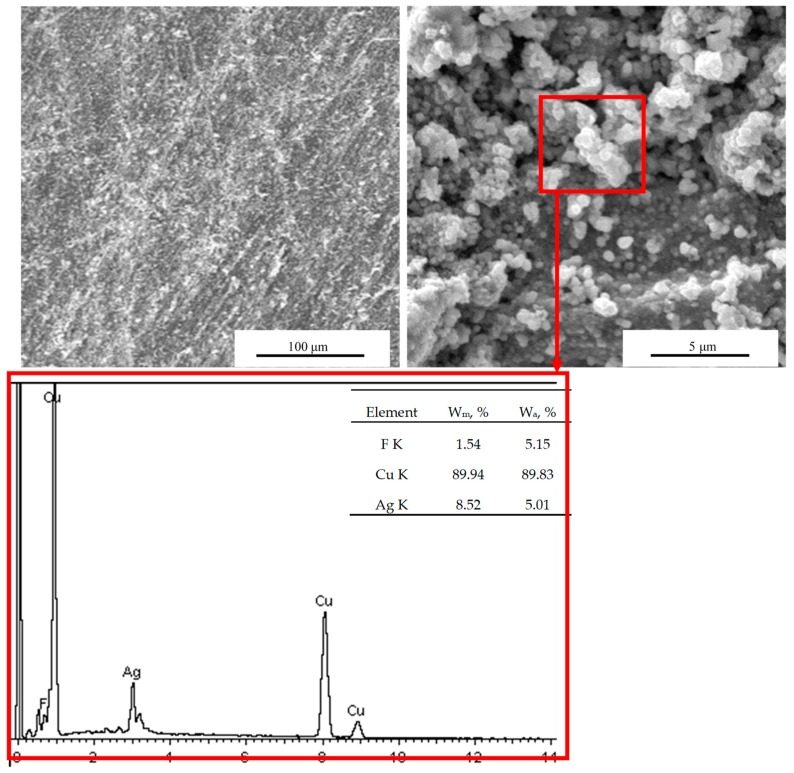
SEM micrographs and EDS spectra of the sample L4.

The surface morphology and EDS values of the Nafion membranes with deposited Cu electrodes are shown in Figure 1. Cu electrodes were deposited on either side of the membranes using a chemical plating method, and the presence of Cu on the surface of the IPMCs was confirmed by EDS measurements. The image of the surface indicates that the Cu coating is relatively uniform and smooth, which suggests good adhesion between the nafion film and the Cu particles. Remarkably, the Cu content revealed by EDS is 89.94% thus confirming that Cu particles were well deposited on the surface of membranes, to ensure a better conductivity and promotes current passage through the IPMCs under the voltage, and improves the actuation performance. It was also found that there was 8.52% silver on the electrode surface, which was mainly due to the reduction of Ag+ to Ag during the process of ion adsorption, which served as the activation nucleation site of copper, which promoted the formation of copper coating, leaving a small amount of silver on the electrode surface. And the sample contains about 1.54% by weight of fluorine from Nafion in Figure 1.

**Figure 2:**
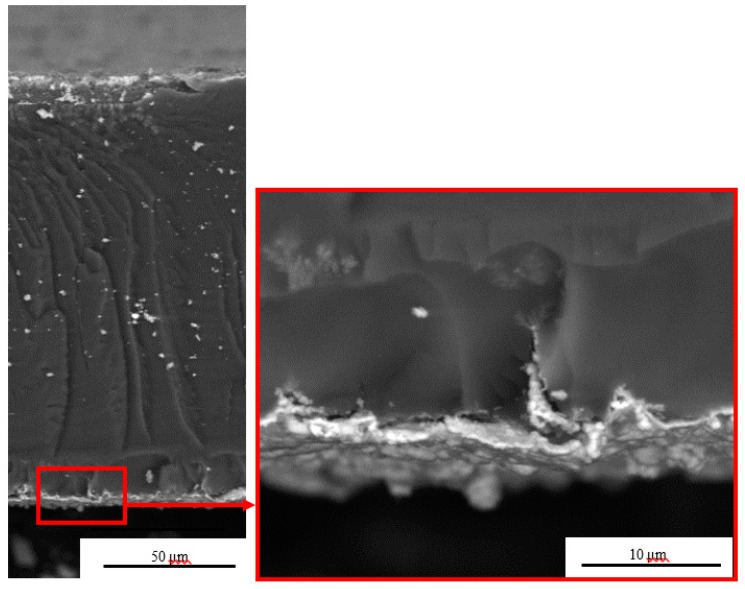
SEM cross-section of the sample (L4).

The cross-sectional SEM images of Cu- IPMCs are shown in Figure 2. The average thickness of the deposited layers of electrodes is approximately 2 μm. The electrodes appear to adhere well on the membrane with no apparent gaps between the electrode and the surface of the membrane. The more the Cu particles deposited on the electrode, the better voltage bias at higher deflections. A needle-like electrodes were formed inside the substrate, and the penetration depth can reach up to around 70 μm. The sandpaper grinding will form a network of scratches on the substrate membrane. These scratches act as favorable sites for nucleation and growth during the chemical deposition of copper, contributing to the formation of needle-like electrodes. Further, around the pit, the Cu particles were squeezed together to form a long continuous conductor poking deep into the polymer. The needle-like structure is very concise without complex branches, which is beneficial for speeding up the actuation and decreasing the stiffness of the material. The white dots in the micrograph are metal particles that have fallen from the electrode layer.

### 3.2. Water Loss Test

**Figure 3:**
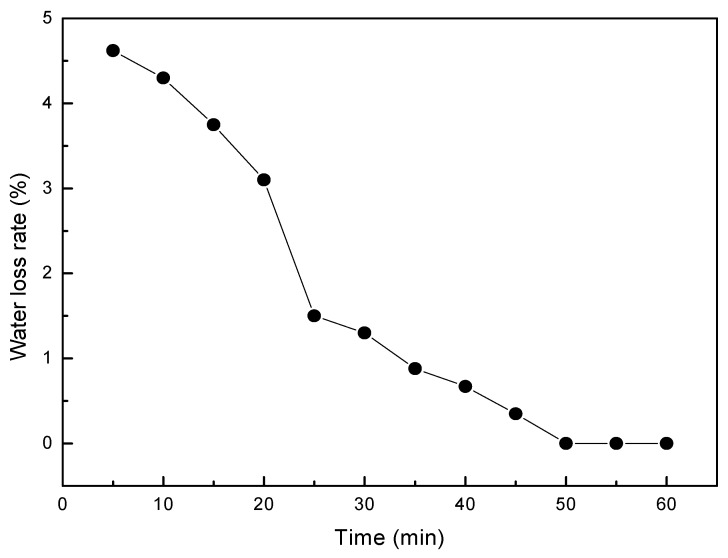
Water loss rate of Cu-IPMC (L4).

The Nafion membrane consists of ionic polymer chains with fixed negatively charged (anionic) groups, between which hydrated positive ions (cations) can migrate. By application of electric field, hydrated cations migrate toward cathode and accumulate near the electrode interface, whereas negative moieties are fixed in the polymer network and cannot compensate volume change due to the accumulation of cation clusters and water electro-osmosis. The water content in the IPMCs is very important for the migration of the hydrated cations, and reducing the migration barrier. However, IPMC loses water during the preparation of electrode and application of the electric field. Figure 3 reveals the relationship between the water loss rate and the time. The water loss decreased with time. The slope of the diagram indicates that the loss of water is fast in the first 25 min, then slows down and does not change after 50 min. Lower water contents in IPMCs limit hydrated cation transfer, thus creating a barrier against migration. So, it is supposed that the IPMC with a low water loss rate will have a fast actuation.

### 3.3. Adhesive Force

**Figure 4:**
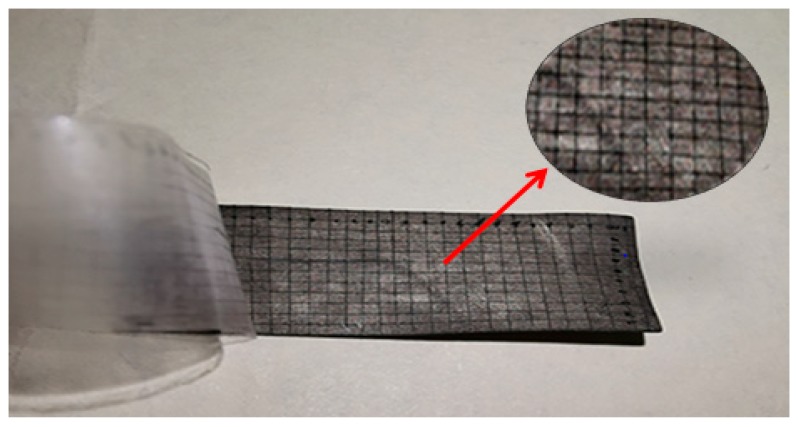
Adhesive force of the sample L4.

The Cu electrodes were formed on the both sides of the membrane by electroless plating, and the electrode formed is known to have good interface. Following to GB9286 test standard, it was determined the adhesion force level at the interface between Cu electrode and the membrane. Figure 4 shows an adhesion comparison before and after. The edge of the cutting line is very smooth, and the square grid is free from any shedding. The copper coating on both sides is closely bonded with the base membrane. The final adhesion level of the sample L4 is 5B with the best performance.

### 3.4. Surface Resistance

**Figure 5:**
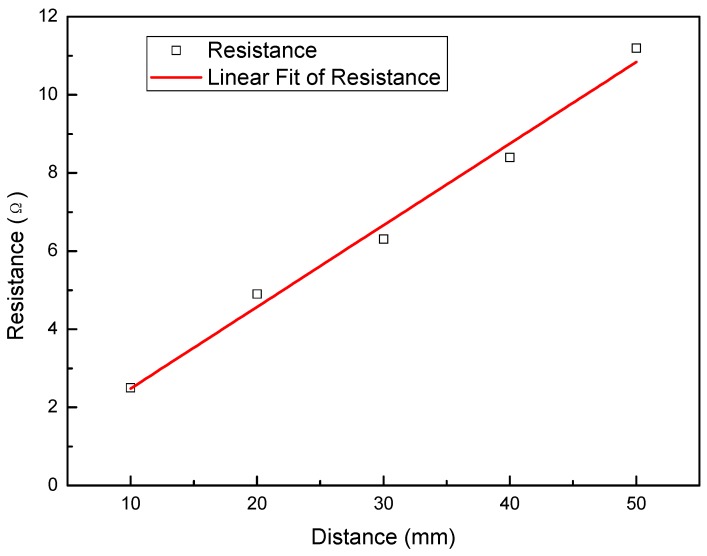
Surface resistances of the IPMC (L4).

Resistance of Cu-IPMC was measured to investigate the uniformity of deposition of the Cu metal electrode layer onto the surface of the Nafion films. The resistance values of IPMC with the length of 50 mm are shown in Figure 5. The average resistance values of IPMCs were obtained to be about 2.5, 4.9, 6.3, 8.4 and 11.2 Ω, respectively. It is observed that the IPMC samples exhibited an increase in the resistance with increasing test distance. And the resistance is 2.1 Ω per centimeter for the L4 sample, indicating the best conductivity.

### 3.5. Effect of IPMC Length on Actuation

#### 3.5.1. Effect of IPMC Length on Displacement

**Figure 6:**
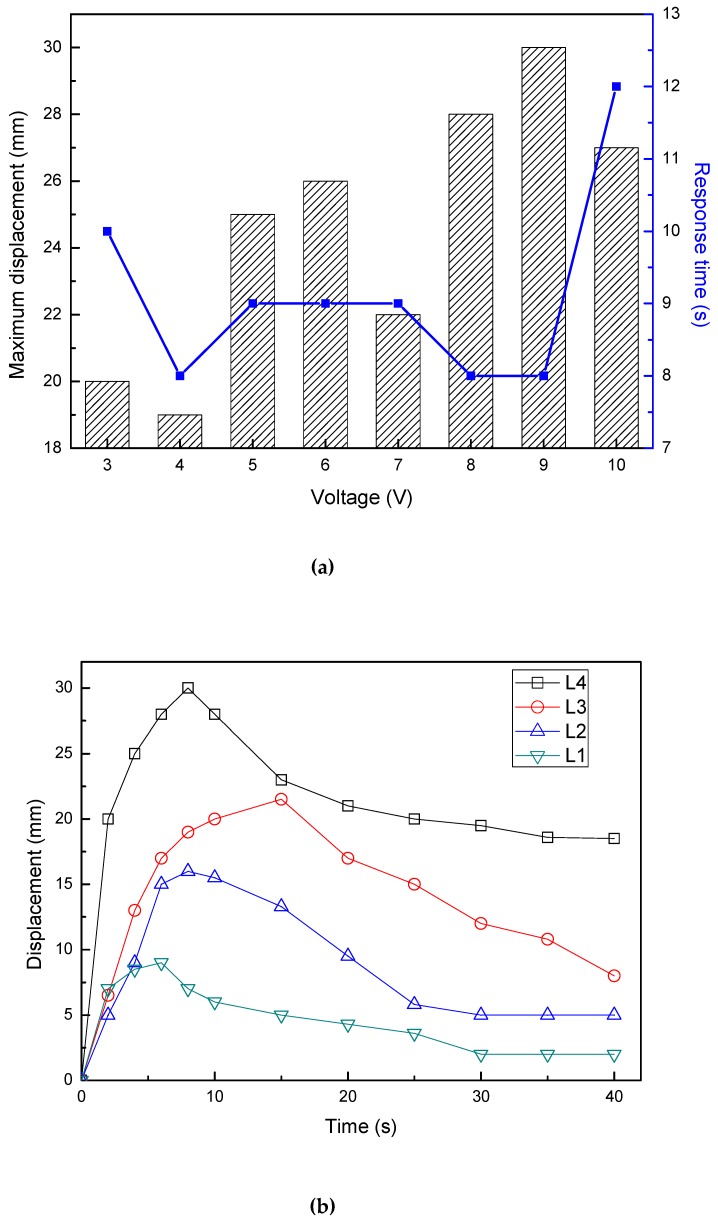
Displacements of the IPMCs with various lengths under different voltages. (**a**) relationships between the maximum displacement or the response time and voltage for L4 sample; (**b**) displacement–time curves of 9 V.

Figure 6 shows the displacement of the IPMCs with various lengths under different voltages. Figure 6a shows the relationship of L4 sample between the maximum displacements or the response time to the maximum displacements and the stimulating voltage. It was found that the maximum displacement increased as the voltage increased, except for 4, 7, and 10 V. However, the response time to the maximum displacement shows no obvious law, basically maintained at 8 s and 9 s. The L4 under voltage of 9 V required 8 s to reach its maximum displacement of 30mm, but the sample required 12 s to achieve a maximum displacement of 27 mm at 10 V. So there is a limit to the applied stimuli voltage, and the drive performance is worse when this critical point is exceeded. Figure 6b reveals the displacement-time curves of the IPMCs with various length under 9 V. The displacement of all samples peaked and then the displacement slowly decreased. The L1 and L4 continuously increased to the maximum displacement of 9 mm at 6 s, and 30 mm at 8 s, respectively. The displacement of the L2 exceeded that of the L1 at about 4 s, and then it continuously increased to the maximum value of 16 mm at 8 s. The L3 increased slowly to reach the maximum displacement of 21.5 mm at 15 s. The actuated displacement increases with the specimen length. When the same displacement was applied and the generated charges were equal, the electrical response was dominated by the length of the IPMC sample.

#### 3.5.2. Effect of IPMC Length on Tip Force

**Figure 7:**
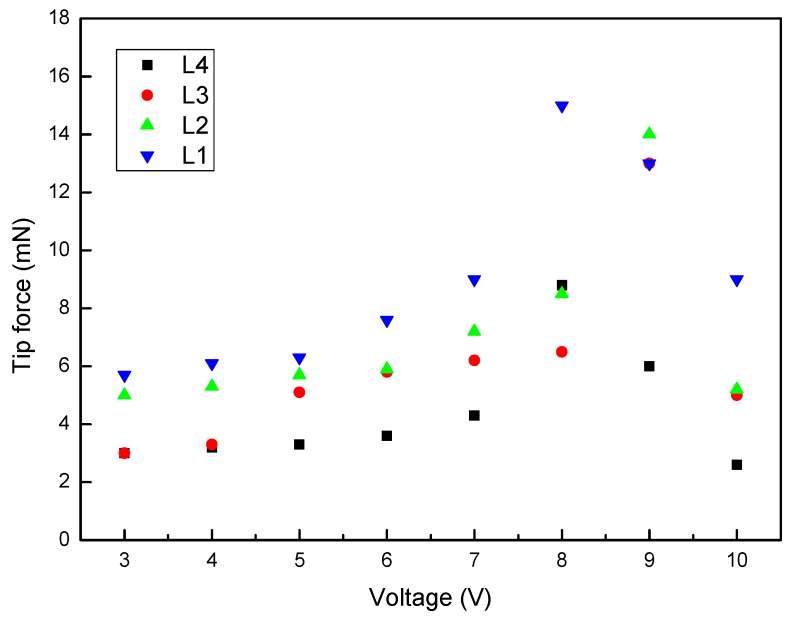
Tip forces of the IPMCs with various lengths under different voltages [28].

Figure 7 shows the tip forces of the IPMCs with various lengths under different voltages. As shown in previous studies [28,29], in general, the tip fore of all samples reached its peak value over a certain time period and then fastly decreased. The L1 and L4 continuously increased to the maximum tip forces of 15 mN and 8.8 mN at 8 V, respectively. Further, the L2 and L3 had maximum tip forces of 14.6 mN and 13 mN at 9 V, respectively. The suitable stimulation voltage for the maximum force output is 8–9 V. In summary, at a fixed voltage, the force output was seen to decrease with the length of the specimen.

### 3.6. Displacements Model and Tip Forces Model.

Experimental data were fitted in order to develop a model able to predict the response of the Cu-IPMC samples. The optimization software was utilized for that purpose. The dimension ratio (DR) and stimulation voltage are taken as two independent variables, while the dependent variables are displacement and force, respectively. If the initial values of unknown parameters are set improperly, the optimization process may hardly converge or even find incorrect results. Therefore, this paper uses the method of Levenberg–Marquardt (LM), starting from any random initial value, and finally finding out the optimal solution by iterative optimization algorithm.

Setting X as the DR of Cu-IPMC, Y as stimulation voltage, *Z_d_* as maximum displacement and *Z_f_* as maximum tip force. The optimal solutions of *Z_d_* and *Z_f_* are obtained from fitting:

(1) $$\begin{array}{l} Z_{d}=5736.77+25.34\times X-10.59\times X^{2}+1.63\times X^{3}\\ \;-17911.25\times \ln Y+21905.67\times {\left(\ln Y\right)}^{2}-13171.12\times {\left(\ln Y\right)}^{3}\\ \;+3898.21\times {\left(\ln Y\right)}^{4}-454.69\times {\left(\ln Y\right)}^{5} \end{array}$$

(2) $$Z_{f}=\frac{41.25-5.00\times X+0.24\times X^{2}-3.00\times Y+0.065\times Y^{2}}{1-0.19\times X+3.16\times Y-0.70\times Y^{2}+0.039\times Y^{3}}$$

**Figure 8:**
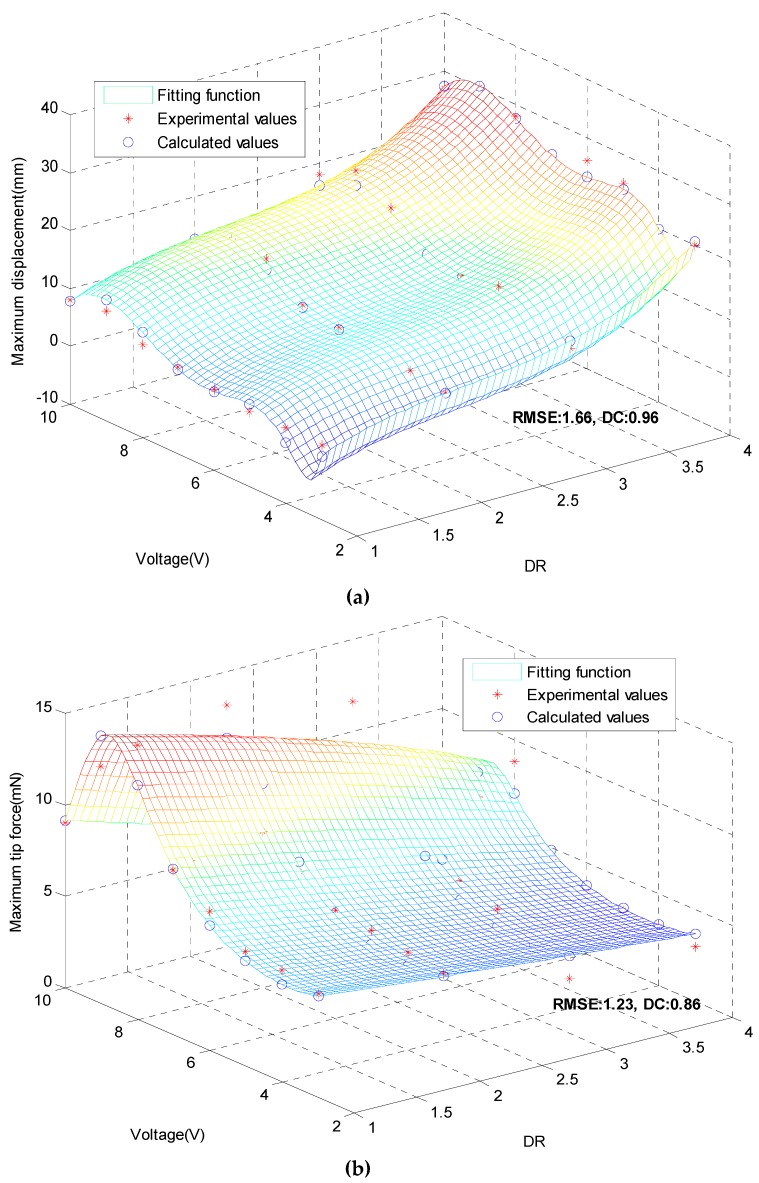
Comparison diagram of experimental and calculated values. (**a**) maximum displacements; (**b**) maximum tip force.

Figure 8 compares the experimental data with the predictions of the fitted model. It can be seen that the experimental data are very close to the calculated values, and the fitted function can characterize the feature points very well, and the data other than the experimental data can be obtained at the same time. RMSE is root of mean square error, DC is determination coefficient in Figure 8. It can also be seen that the RMSEs of the fitted displacement and force function are 1.66 and 1.23, respectively, which indicates that the deviation of the fitting calculation data from the experimental value is small, indicating that the model selection and fitting are ideal, the data prediction is successful, and the method has better accuracy. The DCs of displacement and the force are close to 1 (0.96 and 0.86), confirming the validity of the proposed approach and the ability of regression model to describe the effect of dependency.

In order to analyze in more detail the accuracy of fitting models, four control points were considered. Table 2 compares experimental data with fitted values of maximum displacement and maximum force tip. It can be seen that error exceed 10% only for point 1. The average errors on maximum displacement and maximum tip force are 5.53% and 9.40%, respectively. It can be seen that the model can well present the relationship between the parameters under a certain range and premise, which lays a certain foundation for the further development of Cu-IPMC in the later stage.

## 4. Conclusions

In summary, Cu-IPMCs with various widths were fabricated by the chemical electroless plating methods. And the mechanical and actuation properties of Cu-IPMCs, such as morphology, water loss rate, adhesive force, surface resistance, displacement and tip force were investigated. The average thickness of the deposited Cu layers is approximately 2 μm. Coating is uniform and smoothly deposited with a Cu content of 89.94%. The electrodes adhere well (level of 5B) on the membrane, to ensure a better conductivity and improves the actuation performance. The penetration depth of needle-like electrodes can reach up to around 70 μm. This structure is concise without complex branches, to speed up the actuation. The water content in the IPMCs is very important for the migration of the hydrated cations, and reducing the migration barrier. IPMC with a low water loss rate will have a fast actuation. Overall the maximum displacement increased as the voltage increased, the corresponding response time remained between 8 s and 9 s. The applied voltage for the maximum force output is 8–9 V. Experimental data were fitted by two regression models using the optimization software. This served to explain the actuation properties of Cu-IPMC with various sizes. The present study is a good starting point for the further development of Cu-IPMC.

## Figures and Tables

**Table 1:** Dimensions of the built IPMC specimens.

No.	Thickness (μm)	Width (mm)	Length (mm)	Clamped Part (mm)	Free Part (mm)
L1	187	10	20	10	10
L2	187	10	30	10	20
L3	187	10	40	10	30
L4	187	10	50	10	40

**Table 2:** Comparison of experimental values.and calculated values.

No.	DR	Voltage/V	Displacement			Tip Force		
			Experimental Value/mm	Calculated Value/mm	Error Rate/%	Experimental Value/mN	Calculated Value/mN	Error Rate/%
1	4	6.5	21.50	23.20	7.91	3.80	4.21	10.79
2	4	9.5	28.50	29.90	4.91	4.80	4.34	9.58
3	3	7.3	14.00	13.69	2.21	6.40	7.02	9.69
4	2	7.5	12.00	11.15	7.08	8.60	9.25	7.56
